# Cospeciation of coronavirus and paramyxovirus with their bat hosts in the same geographical areas

**DOI:** 10.1186/s12862-021-01878-7

**Published:** 2021-07-29

**Authors:** Jie Liang, Chunchao Zhu, Libiao Zhang

**Affiliations:** 1 Guangdong Key Laboratory of Animal Conservation and Resource Utilization, Guangdong Public Laboratory of Wild Animal Conservation and Utilization, Institute of Zoology, Guangdong Academy of Sciences, Guangzhou, 510260 China; 2 Zunyi Medical University, Zhuhai Campus, Zhuhai, 519041 China

**Keywords:** Coevolution, Coronavirus, Paramyxovirus, SARS, MERS, Hendra virus, Nipha virus, COVID-19

## Abstract

**Background:**

Bat-borne viruses are relatively host specific. We hypothesize that this host specificity is due to coevolution of the viruses with their hosts. To test this hypothesis, we investigated the coevolution of coronavirus and paramyxovirus with their bat hosts. Published nucleotide sequences of the RNA-dependent RNA polymerase (RdRp) gene of 60 coronavirus strains identified from 37 bat species, the RNA polymerase large (L) gene of 36 paramyxovirus strains from 29 bat species, and the cytochrome B (*cytB*) gene of 35 bat species were analyzed for coevolution signals. Each coevolution signal detected was tested and verified by global-fit cophylogenic analysis using software ParaFit, PACo, and eMPRess.

**Results:**

Significant coevolution signals were detected in coronaviruses and paramyxoviruses and their bat hosts, and closely related bat hosts were found to carry closely related viruses.

**Conclusions:**

Our results suggest that paramyxovirus and coronavirus coevolve with their hosts.

## Background

Bats are reservoirs of many zoonotic viruses, such as members of *Filoviridae* (e.g., Ebola and Marburg viruses), *Paramyxoviridae* (e.g., Hendra and Nipah viruses), and *Coronaviridae (*e.g., severe acute respiratory syndrome coronavirus, Middle East respiratory syndrome coronavirus, severe acute respiratory syndrome coronavirus-2) [1, 2]. Bats live in a wide variety of environments with various feeding habits. They are flying mammals and are effective vehicles for spreading viruses [3].

Coronaviruses are taxonomically placed in the subfamily *Coronavirinae* under the family *Coronaviridae* (International Committee on Taxonomy of Viruses). Bats coronaviruses have been shown to be responsible for the outbreaks of severe acute respiratory syndrome (SARS) in 2002–2003, Middle East respiratory syndrome (MERS) in 2012 [4, 5], and probably the current COVID-19 [2]. They cause serious respiratory and instetinal symptoms with substantial mortality rates [6].

Some paramyxoviruses such as Hendra virus (HeV) and Nipah virus (NiV) are highly pathogenic zoonoses. Both viruses belong to the genus *Henipavirus* of the family *Paramyxoviridae* (International Committee on Taxonomy of Viruses) and have been detected in flying fox bats (*Pteropus *spp.) [7, 8]. *Henipavirus* causes severe symptoms associated with high mortality rates in humans and livestocks. HeV was first detected in Queensland, Australia in 1994, causing acute respiratory disease and febrile illness in horses and humans who have close contact with sick horses [9]. NiV was first detected in Malaysia in 1999 during the outbreak of encephalitis and respiratory illness in pig farmers. In the past few years, sporadic outbreaks of HeV and NiV have occurred in Oceania and Southeast Asia [10–12].

Both coronaviruses and paramyxoviruses have a certain degree of host specificity. Host–parasite specificity has also been observed in malaria parasites [13], bat flies [14], and bacteria [15]. We hypothesized that the host specificity of coronaviruses and paramyxoviruses is a result of co-speciation with their hosts. To test this hypothesis, we compared the nucleotide sequences of the cytochrome B (*cytB*) gene of 61 bat species with those of the RNA dependent RNA polymerase (RdRp) gene of 60 coronavirus strains and the RNA polymerase large (L) gene of 36 paramyxovirus strains.

## Results

### Phylogenetic relationship of bat coronaviruses with their bat hosts

**Fig. 1:**
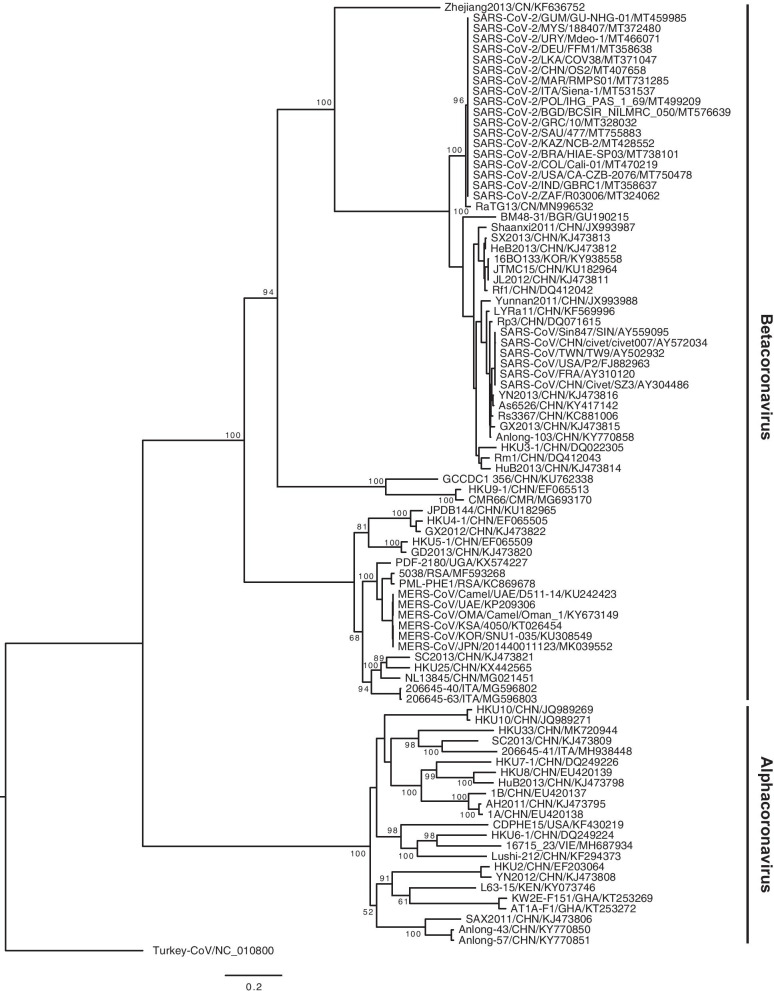
Phylogenetic analysis of the 2734-bp RNA-dependent RNA polymerase (RdRp) gene of coronaviruses from humans and various species of bats

Phylogenetic analyses revealed that the bat coronavirus RaTG13/CN/MN996532 from *Rhinolophus affinis* found in Yunnan Province, China is on the same branch of the phylogenetic tree as the 18 human severe acute respiratory syndrome coronavirus 2 (SARS-CoV-2) strains examined, suggesting that it is a relative of SARS-CoV-2 (Fig. 1). The following 18 bat coronavirus strains are clustered on the same branch of the phylogenetic tree as the six human severe acute respiratory syndrome coronavirus (SARS-CoV) analyzed, suggesting that they are phylogenetic relatives: Rf1/CHN/DQ412042, JTMC15/CHN/KU182964, JL2012/CHN/KJ473811, 16BO133/ROK/KY938558, HeB2013/CHN/KJ473812, SX2013/CHN/KJ473813, Shaanxi2011/CHN/JX993987, Rm1/CHN/DQ412043, HuB2013/CHN/KJ473814, HKU3-1/CHN/DQ022305, Yunnan2011/CHN/JX993988, As6526/CHN/KY417142, YN2013/CHN/KJ473816, Rs3367/CHN/KC881006, GX2013/CHN/KJ473815, Anlong-103/CHN/KY770858, Rp3/CHN/DQ071615, and LYRa11/CHN/KF569996. The strain BM48-31/BGR/GU190215 from *Rhinolophus blasii* living in Bulgaria is found to be distantly related to the six human SARS-CoVs as it is located alone on a branch of the phylogenetic tree (Fig. 1). The following 13 bat coronavirus strains are clustered on the same branch of phylogenetic tree as the six human Middle East respiratory syndrome coronavirus (MERS-CoV) strains examined, suggesting that they are evolutionarily close to each other: JPDB144/CHN/KU182965, HKU4-1/CHN/EF065505, GX2012/CHN/KJ473822, HKU5-1/CHN/EF065509, GD2013/CHN/KJ473820, PDF-2180/UGA/KX574227, 5038/RSA/MF593268, PML-PHE1/RSA/KC869678, SC2013/CHN/KJ473821, HKU25/CHN/KX442565, NL13845/CHN/MG021451, 206645-40/ITA/MG596802, and 206645-63/ITA/MG596803 (Fig. 1).

### Coevolution of bat coronaviruses with their bat hosts

Analyses of all bat *cytB* gene sequences and all bat coronavirus RdRp gene sequences as a whole by the Global test function of software ParaFit and PACo showed evidence of coevolution between bat coronaviruses and their bat hosts (ParaFitGlobal = 390.8896, P = 0.001; m^2^ global value = 57.136, P ≤ 0.001). When each individual sequence was analyzed by the Individual host–parasite (H–P) link test function of ParaFit, 51 of the 60 bat coronavirus strains were found to have a significant coevolution relationship (link) with their bat hosts with a ParaFit1 or ParaFit2 P value ≤ 0.05.

**Fig. 2:**
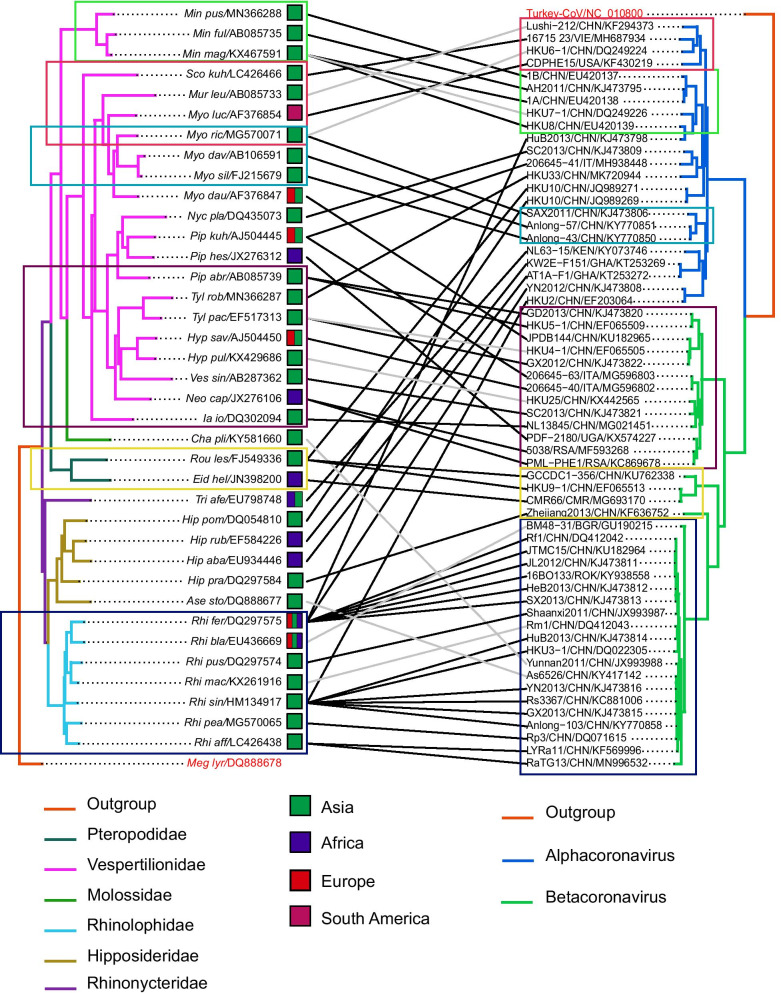
Tanglegram of cophylogenetic relationship between bat hosts and coronaviruses. Black lines denote significant coevolution links between coronaviruses and their hosts (ParaFit tests P ≤ 0.05), and gray lines denote non-significant links. Different groups of coronaviruses and bat species with significant coevolution links are marked with boxes in different colors. Information on host geographical distribution was derived from Simmons (2005)

Bat coronavirus strains examined in this study are divided into alpha and beta groups (Fig. 1). In the alphacoronavirus group, 1B/CHN/EU420137, AH2011/CHN/KJ473795, 1A/CHN/EU420138, HKU7-1/CHN/DQ249226, and HKU8/CHN/EU420139 are related and are all derived from *Miniopterus* bats. Strains SAX2011/CHN/KJ473806, Anlong-57/CHN/KY770851, and Anlong-43/CHN/KY770850 are also related and are all from *Myotis* bats. Similarly, KW2E-F151/GHA/KT253269 and AT1A-F1/GHA/KT253272 are related and are all from *Hipposideros* bats. These observations suggest host specificity of these bat coroanviruses. The following coroanviruses are closely related but are from different species of bats of the same family (*Vespertilionidae*): Lushi-212/CHN/KF294373 (from *Murina leucogaster*), 16715 23/VIE/MH687934 (from *Scotophilus kuhlii*), HKU6-1/CHN/DQ249224 (from *Myotis ricketti*), and CDPHE15/USA/KF430219 (from *Myotis lucifugus*) (Fig. 2). This result suggests that some coronaviruses have a less stringent host specificity than others.

In this study, bat coroanviruses that are related to human SARS-CoVs are referred to as SARSr-CoVs, and those that are related to human MERS-CoVs are referred to as MERSr-CoVs. These viruses are clustered on the betacoronavirus branch of the phylogenetic tree (Fig. 1). Most bat SARSr-CoVs are found in members of the *Rhinolophidae* bat family, and most bat MERSr-CoVs are derived from members of the *Vespertilionidae* bat family. Strains CMR66/CMR/MG693170, HKU9-1/CHN/EF065513, and GCCDC1-356/CHN/KU762338 with 78.03–96.24% RdRp gene sequence identity are clustered together on the same branch of the phylogenetic tree and are all from members of the *Pteropodidae* bat family (Fig. 2).

Strains 206645-40/IT/MG596802 and 206645-63/IT/MG596803 are related with 99.46% identity in RdRp gene sequence and are found in *Hypsugo savii* and *Pipistrelle* *kuhli*, respectively, in Italy (Fig. 2). The SARSr-CoV 16BO133/ROK/KY938558 found in *Rhinolophus ferrumequinum* in South Korea is evolutionarily close to JL2012/KJ473811 (99.71% RdRp sequence identity) and JTMC15/KU182964 (99.68% RdRp sequence identity) found in the same species (*Rhinolophus ferrumequinum*) of bats in Jilin Province, China [16] (Fig. 2). The strain As6526/CHN/KY417142 is found in *Aselliscus stoliczkanus* (family *Hipposideridae*) that are evolutionarily close to *Rhinolophidae* bats (Fig. 2). The strain Yunnan2011/CHN/JX993988 found in Yunnan Province, China is from *Chaerephon plicatus* (family *Molossidae*) bats that are distantly related to *Rhinolophidae* bats (Fig. 2).

### Coevolution of bat paramyxoviruses with their bat hosts

Analyses of all bat *cytB* gene sequences and all paramyxovirus RNA polymerase large (L) gene sequences as a whole by the Global test function of ParaFit and PACo showed evidence of coevolution between bat paramyxoviruses and their bat hosts (ParaFitGlobal = 874.11, P = 0.049; m^2^ global value = 15.49537, P = 0.015). When each individual sequence was analyzed by the Individual H–P link test function of ParaFit, 7 of the 36 bat paramyxovirus strains were found to have a significant host–parasite coevolution relationship (link) with a ParaFit1 or Parafit2 *P* value ≤ 0.05.

**Fig. 3:**
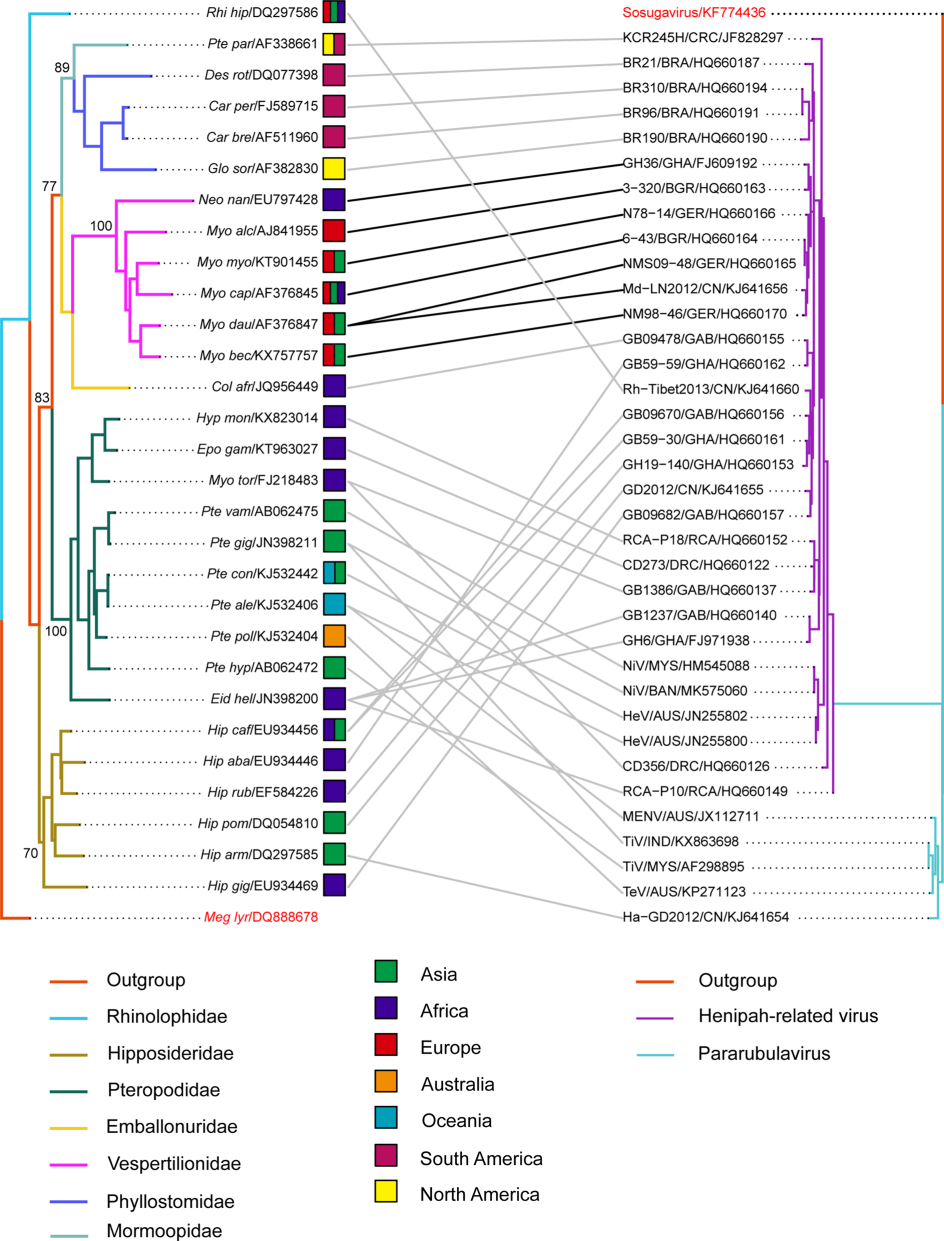
Tanglegram of cophylogenetic relationships between bat hosts and paramyxoviruses. Black lines denote significant coevolution links between paramyxoviruses and their hosts (ParaFit tests P ≤ 0.05), and gray lines denote non-significant links. Different groups of paramyxoviruses and bat species with significant coevolution links are marked with boxes in different colors. Information on host geographical distribution was derived from Simmons (2005)

In this study, we classified unidentified bat paramyxoviruses into four groups PG1–PG4 according to their host speficity. PG1 paramyxovirus strains GB59-59/GHA/HQ660162, GB09670/GAB/HQ660156, GB59-30/GHA/HQ660161, GH19-140/GHA/HQ660153, GD2012/CHN/KJ64165, and GB09682/GAB/HQ660157 are closely related and are all derived from *Hipposideros* bats (family: *Hipposideridae*) (Fig. 3). The following PG2 paramyxovirus strains are form *Pteropodidae* bats and are closely related: RCA-P18/RCA/HQ660152, CD273/DRC/HQ660122, GB1386/GAB/HQ660137, GB1237/GAB/HQ660140, and GH6/GHA/FJ971938 (Fig. 3). KCR245H/CRC/JF828297, BR21/BRA/HQ660187, BR310/BRA/HQ660194, BR310/BRA/HQ660194, and BR190/BRA/HQ660190 that belong to paramyxovirus group 3 (PG3) are closely related (Fig. 3). The host of KCR245H/CRC/JF828297 is *Pteronotus parnellii* bat (family: *Mormoopidae*), and the hosts of the other four strains are bats of the *Pteropodidae* family, including *Desmodus rotundus*, *Carollia perspicillata*, *Carollia brevicauda*, and *Glossophaga soricina* (Fig. 3). Seven closely related strains, including GH36/GHA/FJ609192, 3-320/BGR/HQ660163, N78-14/GER/HQ660166, 6-43/BGR/HQ660164, NMS09-48/GER/HQ660165, Md-LN2012/CHN/KJ641656, and NM98-46/GER/HQ660170 (paramyxovirus group 4, PG4), are found in members of the *Vespertilionidae* bat family (Fig. 3). Identified bat paramyxoviruses including Teviot virus (TeV), Tioman virus (TiV), and Menangle virus (MENV) are members of the genus *Pararubulavir*us; their bat hosts are members of the *Pteropodidae* family. The bat hosts of *Henipavirus*, NiV, and HeV also belong to the *Pteropodidae* family (Fig. 3).

The bat hosts of PG1 paramyxoviruses are distributed mainly in Africa and Asia, and those of PG2 paramyxoviruses are mostly living in Africa. PG3 paramyxoviruses are mostly found in bats in South and North America. The bat hosts of PG4 paramyxoviruses are distributed in Asia, Africa, and Europe. The bat hosts of *Pararubulavirus* and *Hennipahviru*s are found in areas from Asia to Oceania (Fig. 3).

### Event-based cophylogeny

**Fig. 4:**
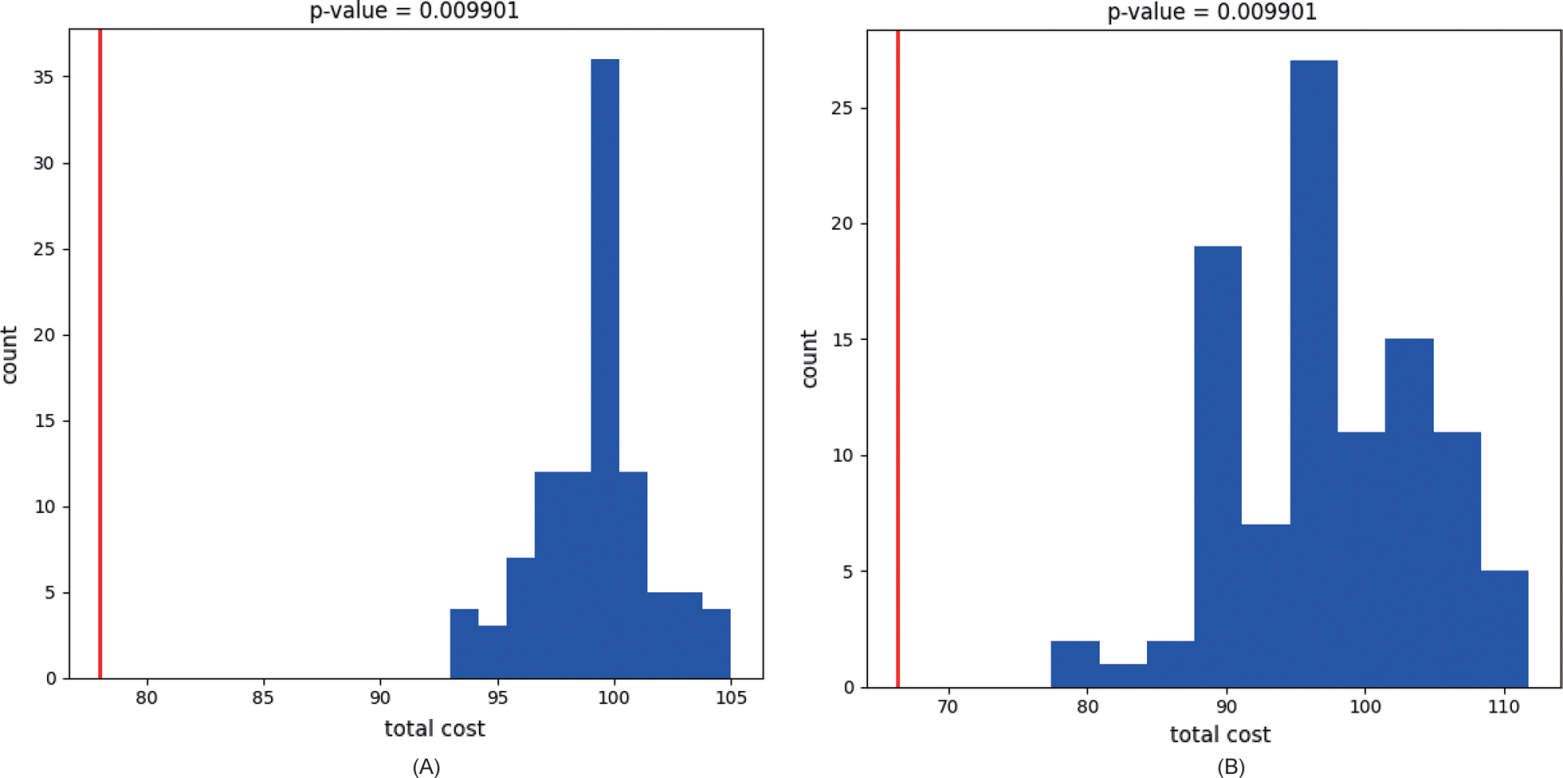
P-value histogram of (**A**) host–coronavirus and (**B**) host–paramyxovirus relationships. The optimal reconciliation cost of the coevolution trees is indicated with a red line, and the optimal cost of the same trees constructed with tip associations permuted at random is shown in blue columns

To confirm the coevolution of coronaviruses and paramyxoviruses with their bat hosts, analyses with the software eMPRess were performed. Results of such analyses refuted the null hypothesis that the host and virus trees and tip associations are formed due to chance at 0.01 level in both host–coronavirus and host–paramyxovirus relationships (P-value = 0.0099). Therefore, we, concluded that these host–virus pairs have coevolved (Fig. 4).

## Disccusion

In this study, we investigated the coevolution relationship between bats and their viral parasites: coronaviruses and paramyxoviruses. These two groups of viruses were chosen because they have been shown to be zoonotic [17]. The sequences of the RNA-dependent RNA polymerase (RdRp) gene of 60 bat coronavirus strains, the RNA polymerase large (L) gene of 36 paramyxovirus strains, and the cytochrome B (cytB) gene of 61 bat species were used to build phylogenetic trees. Cophylo analyses were then performed to determine the relationship between bat genetic trees and those of coronaviruses and paramyxoviruses. Event-based eMPRess test and ParaFit and PACo Global and ParaFit Individual H–P tests were also performed. In eMPRess and Global ParaFit and PACo tests, both groups of the viruses were found to have a significant coevolution relationship with their bat hosts. In ParaFit Individual H–P test, 51 (85%) of the 60 coronavirus strains and 7 (19%) of the 36 paramyxovirus strains had a significant coevolution relationship with their bat hosts.

The construction of cophylogenetic trees revealed that closely related bat coronaviruses are found in closely related bat species (Fig. 2). One example of such observation is that closely related coronavirus strains 1B/CHN/EU420137, AH2011/CHN/KJ473795, 1A/CHN/EU420138, and HKU8/CHN/EU420139 are found in *Miniopterus pusillus*, *Miniopterus fuliginosus*, and *Miniopterus magnater* that are very close to each other. Another example is the coevolution relationship between closely related bats including *Tylonycteris pachypus, Hypsugo savii, Vespertilio sinensis, Neoromicia capensis,* and *Ia io* and the following coronavirus strains: GX2012/CHN/KJ473822, 206645-40/ITA/MG596802, SC2013/CHN/KJ473821, NL13845/CHN/MG021451, 5038/RSA/MF593268, and PML-PHE1/RSA/KC869678. Similar coevolution relationships are found in closely related bat species *Rhinolophus ferrumequinum, Rhinolophus blasii, Rhinolophus pusillus, Rhinolophus macrotis, Rhinolophus sinicus, Rhinolophus pearsonii,* and *Rhinolophus affinis* and the following coronavirus strains: RF1/CHN/DQ412042, JTMC15/CHN/KU182964, JL2012/CHN/KJ473811, 16BO133/ROK/KY938558, HeB2013/CHN/KJ473812, SX2013/CHN/KJ473813, Shaanxi2011/CHN/JX993987, HuB2013/CHK/KJ473814, HKU3-1/CHN/DQ022305, YN2013/CHK/KJ473816, Rs3367/CHN/KC881006, GX2013/CHN/KJ473815, Anlong-103/CHN/KY770858, Rp3/CHN/DQ071615, LYRa11/CHN/KF569996, and RaTG13/CHN/MN996532 (Fig. 2). For paramyxoviruses, closely related bat species *Neoromicia nanus*, *Myotis alcathoe*, *Myotis myotis*, *Myotis capaccinii, Myotis daubentoniid*, and *Myotis bechsteinii* are found to carry the following closely related strains: GH36/GHA/FJ609192, 3-320/BGR/HQ660163, N78-14/GER/HQ660166, 6-43/BGR/HQ660164, NMS09-48/GER/HQ660165, LN2012/CHN/KJ641656, and NM98-46/GER/HQ660170 (Fig. 3).

Results of ParaFit Individual H–P test showed that 85% (51/60) of coronavirus strains but only 19% (7/36) of paramyxovirus strains have a significant coevolution relationship with their bat hosts. Since significant coevolution was found in both groups of the viruses by eMPRess and ParaFit and PACo Global tests, this low positive individual H–P link rate in paramyxoviruses may be due to small sample size.

We postulate that that evolutionary relationship and close habitat of bat species contribute to inter-species transmission of viruses. One observation supporting this hypothesis is that coronavirus strains 206645-40/IT/MG596802 (bat host: *Hypsugo savii*) and 206645-63/IT/MG596803 (bat host: *Pipistrellus kuhlii*) share 99.46% nucleotide sequence identity in their RdRp gene and are found in bats living in the same geographical area, Italy. In addition, coronavirus strains 16BO133/ROK/KY938558, JL2012/KJ473811, and JTMC15/KU182964 are highly related with > 99.6% nucleotide sequence identity in the RdRp gene and are found in bats distributing in areas near each other, including Jilin Province, China (for isolates JL2012/KJ473811 and JTMC15/KU182964) and South Korea (for isolate 16BO133/ROK/KY938558) that is close to China.

Most bat SARSr-CoVs are from *Rhinolophus* bats, but strains As6526/CHN/KY417142 and Yunnan2011/CHN/JX993988 are found in *Aselliscus stoliczkanu* and *Chaerephon plicatus*, respectively*.* Although these two bat species are phylogenetically far apart, they live in the same geographical area, Yunnan Province, China. This observation suggests that distantly related bats in the same geographical location may harbor the same viruses, leading to inter-species transmission of the viruses.

Horseshoe bats are widely distributed in Europe, Asia, Australia, and Africa [18–21] and are potential reservoirs of epidemic coronaviruses [22]. The strain BM48-31/BGR/GU190215 was found in *Rhinolophus blasii* and clustered with other SARSr-CoVs in the coronavirus coevolution tree (Fig. 2). Its relationship with human and civet SARS-CoVs is farther than that of other SARSr-CoVs (Fig. 1), suggesting that they diverged from the same ancestor. Results of previous studies suggest that *Rhinolophus* bats are the natural hosts of human SARS-CoVs [23–25]. Many bat SARSr-CoVs are detected in *Rhinolophus sinicus* and *Rhinolophus ferrumequinum* that distributed in Asia, Africa, and Europe. However, SARSr-CoVs that are highly related to human SARS-CoVs have not been found in *Rhinolophus ferrumequinum* that live in Africa and Europe, probably due to geopgraphical isolation from SARSr-CoVs that are mainly found in Yunnan Province, China [26].

For paramyxoviruses, *Pteropid* bats have been shown to be the natural reservoir of *Henipavirus* [27, 28] and are speculated to be responsible for the outbreaks in Malaysia, Australia, Singapore, Philippine, India, and Bangladesh during the period of 1995–2015 [29–35]. Several species of *Pteropid* bats, including *Pteropus alecto*, *Pteropus conspicillatus*, *Pteropus giganteus*, and *Pteropus vampyrus*, have been found to carry *Henipaviurs.* These bats live in Southeast Asia and Oceania [36], where *Henipavirus* pandemic occurred.

As *Rhinolophus sinicus* bats are found only in China, Nepal, Vietnam, and North India [36] and SARSr-CoVs are mainly found in Yunnan Province, China, SARS-CoV outbreaks have not occurred in other places such as Europe, Africa, Oceania, and America. SARS-CoV-2 is responsible for the COVID-19 pandemic (International Committee on Taxonomy of Viruses). In this study, the strain RaTG13/CHN/MN996532 is found to be very close to SARS-CoV-2 with > 97% nucleotide sequence identity in the RdRp gene and 96% identity at the whole genome level. There are approximately 1100 bases that are different between the genomes of RaTG13/MN996532 and various strains of SARS-CoV-2 suggesting that RaTG13/MN996532 requires at least one intermediate host to transmit to humans [2]. As the host of RaTG13/MN996532 is *Rhinolophus affinis* residing in Yunnan Province, China, it has been speculated that SARS-CoV-2 is derived from *Rhinolophus* bats roosting in areas near Yunnan Province, China, such as Southwest China, Myanmar, Laos, Vietnam, and other Southeast Asian countries [37].

Divergence of bats can be traced back to tens of million years ago [38, 39]. It has been estimated that coronaviruses diverged tens of thousand years ago [40]. This difference may be due to the fact that the genome of coronaviruses is RNA that is more prone to mutations than DNA. It has also been estimated that coronaviruses have been infecting birds and bats for tens of million years, thus providing the opportunity for coevolution with their hosts [41].

## Conclusion

We have found evidence suggesting that both coronavirus and paramyxovirus coevolve with their bat hosts. Understanding the coevolutionary patterns of these viruses with their hosts will allow a better prediction of transmission between bats and humans.

## Methods

### Phylogenetic analysis

The database of Bat-associated Viruses (DBatVir, http://www.mgc.ac.cn/DBatVir/) [42] contains information on various bat-associated viruses, including genome size, lengths of identified genes, date and place (city and country) of isolation, names of the viruses, and GenBank accession numbers of nucleotide sequences of the entire genome or individual genes. With the “browse by virus” function, 60 coronavirus and 36 paramyxovirus strains with all aforementioned information available were found. As this database does not contain actual sequences, nucleotide sequences of selected viral strains were downloaded from the GenBank. These sequences included those of the RNA dependent RNA polymerase (RdRp) gene (2734 bp) of the 60 coronaviruse strains and the RNA polymerase large (L) gene (559 bp) of the 36 paramyxovirus strains. The 60 coronaviruses were derived from 37 different bat species, and the 36 paramyxoviruses were derived from 29 different bat species. Among them, 5 bat species including *Myotis daubentoniid*, *Eidolon helvum*, *Hipposideros abae*, *Hipposideros ruber*, and *Hipposideros pomona* were found to harbor both coronavirus and paramyxovirus. Altogether, 61 different species of bats were identified to be the hosts of the coronavirus and paramyxovirus strains examined in this study. As the cytochrome B (*cytB*) gene is one of the most conserved gene in bats, its sequence was used for coevolution analyses. The *cytB* sequences of 59 of the 61 bat species were downloaded from the GenBank. Since the *cytB* gene sequences of *Miniopterus pusillus* and *Tylonycteris robustula*, that were the hosts of coronavirus strains 1B/CHN/EU420137 and HKU33/CHN/MK720944, respectively, were not available, they were determined in this study. These two bat species were captured from Menghai, Yunnan and Kau O Bat Cave, Macau, respectively. Anal swabs of *Miniopterus pusillus* and a small portion of the patagiums of *Tylonycteris robustula* were obtained. The captured bats were released back to their roosts after sampling. DNA was isolated from these samples and used as the template for amplication by polymerase chain reaction (PCR) of a portion (1140 bp) of the *cytB* gene with primers L14727ag (5′-ATGATATGAAAAACCATCGTTG) and H15915ag (5′-TTTCCNTTTCTGGTTTACAAGAC) [43]. PCR conditions were as follows: 94 °C for 3 min, followed by 20 cycles of 94 °C for 20 s, 46 °C to 52 °C (+ 0.3 °C/cycle) for 30 s, and 72 °C for 90 s and 30 cycles of 94 °C for 20 s, 60 °C for 30 s, 72 °C for 90 s and then maintained at 72 °C for 10 min. The PCR products were sequenced, and the sequences thus obtained have been deposited in the Genbank with accessing numbers MN366287 and MN366288.

To analyze the nucleotide sequences, they were aligned with Clustal Omega (https://www.ebi.ac.uk/Tools/msa/clustalo) [44]. The outgroup sequences included in sequence alignments were those of coronavirus Turkey COV/NC_01080, paramyxovirus Sosugavirus/KF774436, and bat *Megaderma lyra*/DQ888678. Maximum-likelihood phylogenetic trees were constructed using 1000 bootstraps with the raxmlGUI program [45] using the substitution model GTR + I + G, which compares likelihood scores calculated with the jmodeltest 2.1.7 software [46].

### Comparison of host and virus phylogenies

To determine the degree of congruence between bat and virus topologies on phylogenetic trees and identify individual associations contributing to the cophylogenetic relationship the software ParaFit was used [47]. ParaFit tests the null hypothesis that the evolutions of a clade of hosts and a clade of parasites are independent. ParaFit has two types of tests: a global test of coevolution and a test on each host–parasite (H–P) link. The matrices of patristic distances used in global-fit analysis were calculated from the maximum likelihood trees of host and virus phylogenies using the “cophenetic” function of the software package ape in R 3.6.0 [48, 49]. ParaFit analyses were also performed in R with 999 permutations for both global and individual H–P link tests. Each individual host–virus link was considered as significant when its ParaFit 1 or Parafit 2 P-value was ≤ 0.05 [15].

To obtain comparable global goodness-of-fit statistics with Parafit global values, the software package Procrustean Approach to Cophylogeny (PACo) [50] in R was used in conjunction with packages ape and vegan [51]. PACo determines the dependence of one phylogeny upon the other and produces a Procrustes superimposition plot for a graphical assessment of the fit of the parasite phylogeny onto the host phylogeny and a goodness-of-fit statistic. As ParaFit values may be variable, virus matrix in PACo was rotated and scaled to fit the host matrix in order to evaluate the dependence of parasite phylogeny upon host phylogeny. A goodness-of-fit test based on 1000 randomizations was used to assess the significance of such dependence. The associated squared residuals were used to assess the significance of coevolution of each host–virus link [52]. Cophylogenetic trees were generated using the “cophylo” function of the R package phytools [53].

### Even-based cophylogeny

The event-based program eMPRess [54] was used to determine whether the pairs of coronavirus, paramyxovirus, and their hosts coevolve with each other. eMPRess is a tool for reconciling pairs of phylogenetic trees based on the Duplication-Transfer-Loss (DTL) model [55], which is performed using a maximum parsimony formulation to determine the associated cost of each coevolution event. eMPRess computes and displays the distances between every pair of maximum parsimony reconciliation (MPR). The distance between two MPRs is the number of events in one MPR. The maximum likelihood (ML) trees of hosts and viruses were used as inputs, and the analysis was conducted using the following eMPRess parameters: duplication cost = 1, transfer cost = 2, and loss cost = 1. The optimal reconciliation cost for each dataset was compared with that of the same tree with tip associations permuted at random.

## Data Availability

The nucleotide sequences of the *cytB* gene of *Miniopterus pusillus* and *Tylonycteris robustula* bats are available at GenBank with accession numbers MN366287 and MN366288.

## Abbreviations

RdRp: RNA-dependent RNA polymerase
L: RNA polymerase large gene
*cytB*: Cytochrome B
HeV: Hendra virus
NiV: Nipah virus
TeV: Teviot virus
TiV: Tioman virus
MENV: Menangle virus
H–P: Host–parasite
SARS-CoV-2: Severe acute respiratory syndrome coronavirus 2
SARS-CoV: Severe acute respiratory syndrome coronavirus
MERS-CoV: Middle East respiratory syndrome coronavirus
SARSr-CoV: Bat coronavirus related to human SARS-CoV
MERSr-CoV: Bat coronavirus related to human MERS-CoV

## References

[1] Luis AD, Hayman DT, O'Shea TJ, Cryan PM, Gilbert AT, Pulliam JR (2013). A comparison of bats and rodents as reservoirs of zoonotic viruses: are bats special?. Proc Biol Sci.

[2] Zhou P, Yang XL, Wang XG, Hu B, Zhang L, Zhang W (2020). A pneumonia outbreak associated with a new coronavirus of probable bat origin. Nature.

[3] Serra-Cobo J, López-Roig M (2017). Bats and emerging infections: an ecological and virological puzzle. Adv Exp Med Biol.

[4] Zaki AM, van Boheemen S, Bestebroer TM, Osterhaus AD, Fouchier RA (2012). Isolation of a novel coronavirus from a man with pneumonia in Saudi Arabia. N Engl J Med.

[5] Drexler JF, Corman VM, Drosten C (2014). Ecology, evolution and classification of bat coronaviruses in the aftermath of SARS. Antiviral Res.

[6] Gralinski LE, Baric RS (2015). Molecular pathology of emerging coronavirus infections. J Pathol.

[7] Smith I, Broos A, de Jong C, Zeddeman A, Smith C, Smith G (2011). Identifying Hendra virus diversity in pteropid bats. PLoS ONE.

[8] Anderson DE, Islam A, Crameri G, Todd S, Islam A, Khan SU (2019). Isolation and full-genome characterization of Nipah viruses from bats, Bangladesh. Emerg Infect Dis.

[9] Selvey LA, Wells RM, McCormack JG, Ansford AJ, Murray K, Rogers RJ (1995). Infection of humans and horses by a newly described morbillivirus. Med J Aust.

[10] Harit AK, Ichhpujani RL, Gupta S, Gill KS, Lal S, Ganguly NK (2006). Nipah/Hendra virus outbreak in Siliguri, West Bengal, India in 2001. Indian J Med Res.

[11] Mahalingam S, Herrero LJ, Playford EG, Spann K, Herring B, Rolph MS (2012). Hendra virus: an emerging paramyxovirus in Australia. Lancet Infect Dis.

[12] Sharma V, Kaushik S, Kumar R, Yadav JP, Kaushik S (2019). Emerging trends of Nipah virus: a review. Rev Med Virol.

[13] Ricklefs RE, Fallon SM, Bermingham E (2004). Evolutionary relationships, cospeciation, and host switching in avian malaria parasites. Syst Biol.

[14] Nikon N, Sato M, Kondo N, Fukatsu T (2011). Phylogenetic comparison between nycteribiid bat flies and their host bats. Med Entomol Zool.

[15] Lei BR, Olival KJ (2014). Contrasting patterns in mammal–bacteria coevolution: bartonella and leptospira in bats and rodents. PLoS Negl Trop Dis.

[16] Xu L, Zhang F, Yang W, Jiang T, Lu G, He B (2016). Detection and characterization of diverse alpha- and betacoronaviruses from bats in China. Virol Sin.

[17] Rizzo F, Edenborough KM, Toffoli R, Culasso P, Zoppi S, Dondo A (2017). Coronavirus and paramyxovirus in bats from Northwest Italy. BMC Vet Res.

[18] DeBlase AF, Martin RL (1973). Distributional notes on bats (Chiroptera: Rhinolophidae, Vespertilionidae) from Turkey. Mammalia.

[19] Krystufek B (1993). Geographic variation in the greater horseshoe bat *Rhinolophus ferrumequinum* in south-eastern Europe. Acta Theriol.

[20] Maree S, Grant WS (1997). Origins of horseshoe bats (Rhinolophus, Rhinolophidae) in southern Africa: evidence from allozyme variability. J Mamm Evol.

[21] Hand S, Kirsch AW, Kunz TH, Racey PA (1998). A southern origin for the Hipposieridae (Microchiroptera)? Evidence from the Australian fossil record. Bat biology and conservation.

[22] Lau SKP, Wong ACP, Zhang L, Luk HKH, Kwok JSL, Ahmed SS (2019). Novel bat alphacoronaviruses in southern China support Chinese horseshoe bats as an important reservoir for potential novel coronaviruses. Viruses.

[23] Lau SK, Woo PC, Li KS, Huang Y, Tsoi HW, Wong BH (2005). Severe acute respiratory syndrome coronavirus-like virus in Chinese horseshoe bats. Proc Natl Acad Sci USA.

[24] Ge XY, Li JL, Yang XL, Chmura AA, Zhu G, Epstein JH (2013). Isolation and characterization of a bat SARS-like coronavirus that uses the ACE2 receptor. Nature.

[25] Yang XL, Hu B, Wang B, Wang MN, Zhang Q, Zhang W (2015). Isolation and characterization of a novel bat coronavirus closely related to the direct progenitor of severe acute respiratory syndrome coronavirus. J Virol.

[26] Hu B, Zeng LP, Yang XL, Ge XY, Zhang W, Li B (2017). Discovery of a rich gene pool of bat SARSr-coronaviruses provides new insights into the origin of SARS coronavirus. PLoS Pathog.

[27] Young PL, Halpin K, Selleck PW, Field H, Gravel JL, Kelly MA (1996). Serologic Evidence for the presence in Pteropus bats of a paramyxovirus related to equine morbillivirus. Emerg Infect Dis.

[28] Halpin K, Young PL, Field HE, Mackenzie JS (2000). Isolation of Hendra virus from pteropid bats: a natural reservoir of Hendra virus. J Gen Virol.

[29] Murray K, Selleck P, Hooper P, Hyatt A, Gould A, Gleeson L (1995). A morbillivirus that caused fatal disease in horses and humans. Science.

[30] Chua KB, Goh KJ, Wong KT, Kamarulzaman A, Tan PS, Ksiazek TG (1999). Fatal encephalitis due to Nipah virus among pig-farmers in Malaysia. Lancet.

[31] Chua KB, Bellini WJ, Rota PA, Harcourt BH, Tamin A, Lam SK (2000). Nipah virus: a recently emergent deadly paramyxovirus. Science.

[32] Hsu VP, Hossain MJ, Parashar UD, Ali MM, Ksiazek TG, Kuzmin I (2004). Nipah virus encephalitis re-emergence, Bangladesh. Emerg Infect Dis.

[33] Chadha MS, Comer JA, Lowe L, Rota PA, Rollin PE, Bellini WJ, Ksiazek TG (2006). Nipah virus-associated encephalitis outbreak, Siliguri, India. Emerg Infect Dis.

[34] Arankalle VA, Bandyopadhyay BT, Ramdasi AY, Jadi R, Patil DR, Rahman M (2011). Genomic characterization of Nipah virus, West Bengal, India. Emerg Infect Dis.

[35] Ching PK, de los Reyes VC, Sucaldito MN, Tayag E, Columna-Vingno AB, Malbas FF (2015). Outbreak of henipavirus infection, Philippines. Emerg Infect Dis.

[36] Simmons NB, Wilson D, Reeder D (2005). Order Chiroptera. Mammal species of the world: a taxonomic and geographic reference.

[37] Latinne A, Hu B, Olival KJ, Zhu G, Zhang L, Li H, Daszak P (2020). Origin and cross-species transmission of bat coronaviruses in China. Nat Commun.

[38] Teeling EC, Springer MS, Madsen O, Bates P, O’brien SJ, Murphy WJ (2005). A molecular phylogeny for bats illuminates biogeography and the fossil record. Science.

[39] Agnarsson I, Zambrana-Torrelio CM, Flores-Saldana NP, May-Collado LJ (2011). A time-calibrated species-level phylogeny of bats (Chiroptera, Mammalia). PLoS Curr.

[40] Woo PC, Lau SK, Lam CS, Lau CC, Tsang AK, Lau JH (2012). Discovery of seven novel Mammalian and avian coronaviruses in the genus deltacoronavirus supports bat coronaviruses as the gene source of alphacoronavirus and betacoronavirus and avian coronaviruses as the gene source of gammacoronavirus and deltacoronavirus. J Virol.

[41] Wertheim JO, Chu DK, Peiris JS, Kosakovsky Pond SL, Poon LL (2013). A case for the ancient origin of coronaviruses. J Virol.

[42] Chen L, Liu B, Yang J, Jin Q (2014). DBatVir: the database of bat-associated viruses. Database (Oxford).

[43] Guillén-Servent A, Francis CM (2006). A new species of bat of the Hipposideros bicolor group (Chiroptera: Hipposideridae) from Central Laos, with evidence of convergent evolution with Sundaic taxa. Acta Chiropterol.

[44] Sievers F, Higgins DG (2014). Clustal Omega, accurate alignment of very large numbers of sequences. Methods Mol Biol.

[45] Silvestro D, Michalak I (2012). raxmlGUI: a graphical front-end for RAxML. Organ Diver Evol.

[46] Darriba D, Taboada GL, Doallo R, Posada D (2012). Jmodeltest 2: more models, new heuristics and parallel computing. Nat Methods.

[47] Legendre P, Desdevises Y, Bazin E (2002). A statistical test for host–parasite coevolution. Syst Biol.

[48] Paradis E, Claude J, Strimmer K (2004). Ape: analyses of phylogenetics and evolution in R language. Bioinformatics.

[49] R Core Team. R: a language and environment for statistical computing. Vienna, Austria: R Foundation for Statistical Computing. 2016. https://www.R-project.org/.

[50] Balbuena JA, Míguez-Lozano R, Blasco-Costa I (2013). Paco: a novel procrustes application to cophylogenetic analysis. PLoS ONE.

[51] Oksanen J, Blanchet FG, Kindt R, Legendre P, Minchin PR, O’Hara RB, et al. Vegan: Community Ecology Package. 2016. https://CRAN.R-project.org/package=vegan.

[52] Singh G, Grande FD, Divakar PK, Otte J, Crespo A, Schmitt I (2017). Fungal–algal association patterns in lichen symbiosis linked to macroclimate. New Phytol.

[53] Revell LJ (2011). Phytools: an R package for phylogenetic comparative biology (and other things). Methods Ecol Evol.

[54] Santichaivekin S, Yang Q, Liu JY, Mawhorter R, Jiang J, Wesley T, et al. eMPRess: a systematic cophylogeny reconciliation tool. Bioinformatics. 2020;betaa978. 10.1093/bioinformatics/btaa978. 10.1093/bioinformatics/btaa978 33216126

[55] Ma W, Smirnov D, Libeskind-Hadas R. DTL reconciliation repair. BMC Bioinformatics. 2017;18(Suppl 3):76. 10.1186/s12859-017-1463-9 PMC5374596 28361686

